# Comparison of Retinal Metabolic Activity and Structural Development between rd10 Mice and Normal Mice Using Multiphoton Fluorescence Lifetime Imaging Microscopy

**DOI:** 10.3390/cimb46010039

**Published:** 2024-01-06

**Authors:** Erin Su, Niranjana Kesavamoorthy, Jason A. Junge, Mengmei Zheng, Cheryl Mae Craft, Hossein Ameri

**Affiliations:** 1 Department of Ophthalmology, USC Roski Eye Institute, Keck School of Medicine, University of Southern California, Los Angeles, CA 90033, USA; erinsu@usc.edu (E.S.); niranjana.kesavamoorthy@med.usc.edu (N.K.); ccraft@usc.edu (C.M.C.); 2 Department of Biological Sciences, David Dornsife College of Letters Arts and Sciences, University of Southern California Dana, Los Angeles, CA 90089, USA; junge@usc.edu

**Keywords:** retina, multiphoton fluorescence lifetime imaging microscopy, FLIM, NAD(P)H, glycolysis, oxidative phosphorylation, retinal degeneration, retinal development

## Abstract

Fluorescence lifetime imaging microscopy (FLIM) is a technique that analyzes the metabolic state of tissues based on the spatial distribution of fluorescence lifetimes of certain interacting molecules. We used multiphoton FLIM to study the metabolic state of developing C57BL6/J and rd10 retinas based on the fluorescence lifetimes of free versus bound nicotinamide adenine dinucleotide and nicotinamide adenine dinucleotide phosphate (NAD(P)H), with free NAD(P)H percentages suggesting increased glycolysis and bound NAD(P)H percentages indicating oxidative phosphorylation. The mice were sacrificed and enucleated at various time points throughout their first 3 months of life. The isolated eyecups were fixed, sectioned using a polyacrylamide gel embedding technique, and then analyzed with FLIM. The results suggested that in both C57BL6/J mice and rd10 mice, oxidative phosphorylation initially decreased and then increased, plateauing over time. This trend, however, was accelerated in rd10 mice, with its turning point occurring at p10 versus the p30 turning point in C57BL6/J mice. There was also a noticeable difference in oxidative phosphorylation rates between the outer and inner retinas in both strains, with greater oxidative phosphorylation present in the latter. A greater understanding of rd10 and WT metabolic changes during retinal development may provide deeper insights into retinal degeneration and facilitate the development of future treatments.

## 1. Introduction

Inherited retinal degenerations are among the most debilitating causes of blindness. Though they are currently incurable, an improved understanding of retinal development and its underlying metabolic processes may facilitate future treatment advancements. Various mouse models have been used to further study retinal degeneration, including the rd10 mouse model, which encodes a missense point mutation in Pde6b (cGMP phosphodiesterase 6B; rod receptor; beta polypeptide). This phenotype has been shown to exhibit progressive rod cell death starting around postnatal (P) day 16–18 and is considered an ideal drug therapy model for human retinitis pigmentosa [1,2,3]. Wild-type (WT) versus rd10 development has been previously studied, especially from a structural and functional standpoint; however, similar metabolic studies performed over time have remained limited, despite increasing evidence that retinal metabolic changes may be contributory to progressive photoreceptor loss [2,3,4,5].

Fluorescence lifetime imaging microscopy (FLIM) shows the fluorescence lifetimes of various molecules, a property that is independent of fluorophore concentration and instead is based on molecules’ local interactions [6]. By using a FLIM multiphoton approach versus the confocal method, light can penetrate more deeply and specifically to generate an optical section of the sample at the desired plane of focus [7]. Certain metabolism-related fluorophores, including free and bound reduced nicotinamide adenine dinucleotide and nicotinamide adenine dinucleotide phosphate (NAD(P)H), have characteristic fluorescence lifetimes [6]. Free NAD(P)H has a shorter lifetime than bound NAD(P)H, with the former signaling glycolysis, the latter oxidative phosphorylation (OXPHOS), and the relative shifts between them indicating the exchanging of electrons [6,8]. Together, their relative fluorescence lifetimes can be studied in different conditions and used to show cellular metabolic states in various tissues, including in human organoids, mouse oocytes, kidneys, and eyes [6,8,9,10,11,12,13,14,15]. These metabolic shifts can also be helpful when assessing the degree of oxidative stress [11]. When applied in this setting, increased free NAD(P)H indicates more glycolysis and potentially increased oxidative stress. However, increased NAD(P)H cannot be the sole determinant of a tissue’s overall state of health and must be assessed within the context of any metabolic changes.

To date, FLIM eye studies have mostly been performed on healthy, functional eyes [12,14]. Of the ocular pathologies investigated so far, FLIM has been performed on eyes with age-related macular degeneration (AMD) to investigate retinal pigment epithelium (RPE) and sub-RPE deposits [16]. To the best of our knowledge, there have not been other peer-reviewed FLIM articles studying inherited retinal degeneration, though this topic, as well as other ocular pathologies, like hydroxychloroquine retinopathy and AMD, have been explored using fluorescence lifetime imaging ophthalmoscopy (FLIO), a similar imaging modality [17,18]. In this study, we used FLIM to investigate retinal development and metabolic changes in WT and rd10 mice in their first three months of life with the goal of better understanding the metabolic changes that occur during retinal development in both healthy and inherited retinal degeneration eye models. While structural changes, most notably retinal thinning and degeneration, have been well established in rd10 mice, metabolic changes throughout retinal development have not been well documented. In this study, we hope to confirm previous structural findings on WT and rd10 retinal development, as well as investigate metabolic shifts and the usage of glycolysis versus OXPHOS during this time.

## 2. Materials and Methods

WT (C57BL6/J) and rd10 (*Pde6b^rd1-J^*) mice were purchased from Jackson Laboratory (Bar Harbor, ME, USA) and housed in a 12-h light/dark cycle, with food and water provided ad libitum. All procedures were compliant with the Institutional Animal Care and Use Committee (IACUC) guidelines at the University of Southern California.

Animals from both the WT and rd10 groups were euthanized and then enucleated at certain time points on postnatal (P) days 0, 5, 10, 15, 20, 30, 56, and 84. The P0 timepoint was chosen to capture the structural and metabolic state at birth for both strains. P5–15 were chosen to investigate any potential interim structural or metabolic changes before the P16–18 period, a time when rd10 mice begin to show rod cell death. P20 and P30 were chosen to capture the initial, faster phase of photoreceptor degeneration in rd10 mice, and the P56 and P84 timepoints were chosen to show that rd10 retinal thinning slows dramatically after P30 [4]. In total, 121 eyes were included in this study. Tissue preparation was performed as previously described [14]. Cautery was used to mark the superior aspect of each cornea before enucleation to preserve eyeball orientation. The corneas were punctured prior to undergoing corneal, iris, and lens removal. The eyeballs were allowed to soak in 4% paraformaldehyde between each removal step and were soaked for a total of 3 h. After the eyecups were isolated, they were embedded in a polyacrylamide gel made up of 3 mL of 40% bisacrylamide, 1 mL of 10× Tris-buffered saline, 6 mL of ultrapure Milli-Q water, 47 μL of 10% ammonium persulfate, and 30 μL of tetramethyl ethylenediamine based on Hayaran et al.’s established method [19]. The Leica Microtome VT1200 (Wetzlar, Germany) was used to create 100 μm-thick sections of the embedded eyecups. The sections created were either superior–inferior or nasal–temporal sections. The central-most sections, which included the peripheral retina and optic nerve, were selected for final imaging and analysis.

**Figure 1:**
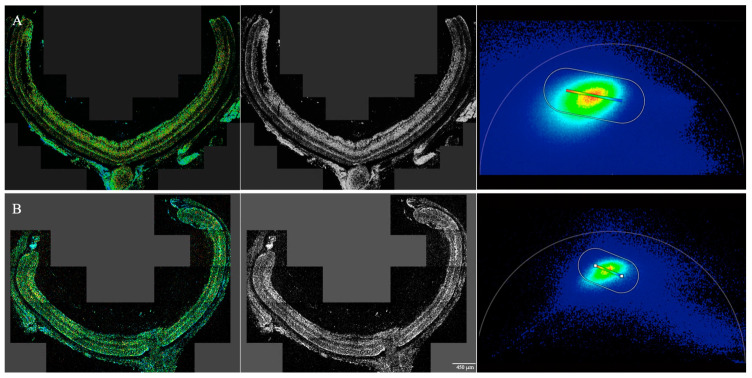
NAD(P)H analysis of the wild-type (WT) and rd10 mice retina at P10. (**A**) WT mouse retina. Left: Representative image of the entire retina in the NAD(P)H channel. Middle: Autofluorescent image. Right: Phasor plot indicating the NAD(P)H signal for the WT specimen image. The unit circle is the NAD(P)H signal, with its concentric colors indicating signal strength, with red being the strongest. A metabolic trajectory was then drawn from the edges of the unit circle passing through the center of mass using the ratiometric analysis tool, with the color bar marked on the top of the trajectory to show bound and free NAD(P)H, with the redder end of the spectrum indicating more bound NAD(P)H and increased OXPHOS. (**B**) rd10 mice retina. Left: Representative image of the entire retina. Middle: Autofluorescent image of rd10 at P10. Right: Phasor plot indicating the NAD(P)H signal for the rd10 specimen image, showing a rainbow spectrum with a similar metabolic trajectory as drawn above. As evidenced above, there is no significant difference between the WT and rd10 mice at the P10 time point, both showing colors in the yellow and green ranges of the spectrum on the NAD(P)H channel, therefore showing a moderate amount of bound NAD(P)H and OXPHOS at this time. Scale bar: 450 μm.

The Leica SP9 DIVE falcon (Wetzlar, Germany) with a Leica 25×/0.95 Na CSII water immersion objective, multiphoton excitation at 740 nm, and a 425–475 nm detection band were used for the autofluorescent metabolic imaging of NAD(P)H (Figure 1). The complex wavelet filter was applied to the phasor data [20]. The excitation and emission spectra were chosen based on our previous protocol and that of others [10,14,21,22,23].

Superior–inferior sections were divided into superior and inferior regions, and nasal–temporal sections were divided into nasal and temporal regions. Each region was divided into three zones: “posterior” (400 μm from the optic disc), far periphery (near the ora serrata), and mid-periphery (halfway between the optic disk and ora serrata). Each zone was divided into outer (from the photoreceptor outer segment to the outer nuclear layer) and inner (outer plexiform layer to the retinal nerve fiber layer) sections. Using Leica’s LASX FLIM FCS software version 4.5 (Wetzlar, Germany), we performed phasor analysis by creating standardized 150 μm-wide regions of interest (ROIs) and calculating the position of each ROI’s center of mass along the pre-drawn metabolic trajectory to calculate the ratio of bound NAD(P)H/total NAD(P)H with the ratiometric analysis tool.

SPSS and Excel were used for statistical analysis. For the WT and rd10 mice, the mean and standard deviation (SD) of the percentage of bound NAD(P)H were calculated for each timepoint for the overall (all retinal layers, meaning the outer and inner layers), outer, and inner retinas in the posterior, mid-periphery, and peripheral zones. ANOVA was used to compare the percentage of bound NAD(P)H over time for both mouse strains across the chosen time points, and additional Tukey post-hoc tests were used to compare the percentage of bound NAD(P)H between each time point to determine the statistical significance at each point in time. Independent *t*-tests were used to compare bound NAD(P)H percentages between the WT and rd10 time points. To account for multiple comparisons, we applied a Bonferroni correction by dividing the desired alpha level of 0.05 by the number of comparisons being made.

## 3. Results

**Figure 2:**
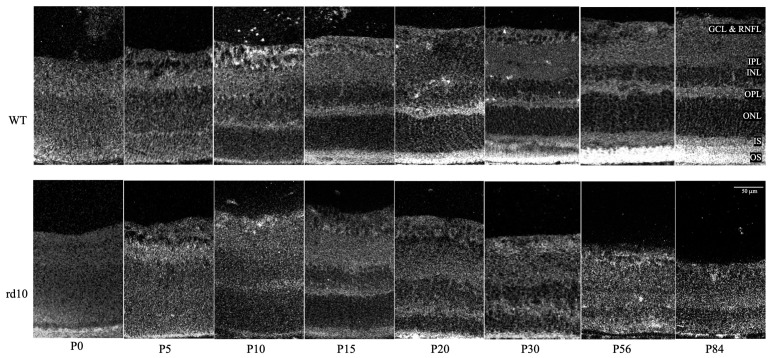
Autofluorescent images of retinal sections showing wild-type (WT) and rd10 early retinal development. Retinal layers were fully differentiable by P10 in both strains and remained morphologically similar up to P15. After P15, rd10 retinas showed progressive thinning, mainly in the outer layers. After P30, the rd10 retinas showed blurred boundaries between the different retinal layers and were harder to distinguish versus the WT retinas. The retinal sections that are shown measure 150 μm across. GCL and RNFL—ganglion cell layer and retinal nerve fiber layer, IPL—inner plexiform layer, INL—inner nuclear layer, OPL—outer plexiform layer, ONL—outer nuclear layer, IS—photoreceptor inner segment, and OS—photoreceptor outer segment. The outer retinal region extends from the OS to the ONL, while the inner retinal region extends from the OPL to the RNFL. The scale bar shown is applicable to all the above images.

WT and rd10 mice were imaged at eight time points over the first 3 months of life (Figure 2). On autofluorescence imaging of retinal sections, WT and rd10 mice appeared to be similar until P15; afterward, while the WT mice showed further growth of the inner and outer segments of photoreceptors, the rd10 mice exhibited degeneration and progressive thinning of the outer nuclear layer and inner and outer segments of photoreceptors, which disappeared at P56. Additionally, at later time points, in the rd10 mice, retinal layers were more difficult to discern compared to their WT counterparts.

The percentage of bound NAD(P)H was measured in both WT and rd10 mice in all time points across the bilateral posterior, mid-peripheral, and far peripheral zones in the overall retina and measured from P10 onwards in the outer retina (spanning from the outer nuclear layer to the outer segment of photoreceptors) and inner retina (spanning from the retinal nerve fiber layer to the outer plexiform layer). For both strains in all the retinal layers, bound NAD(P)H percentages were shown to significantly change over time (WT overall retina *p* < 0.001; rd10 overall retina *p* < 0.001; WT outer retina *p* = 0.001; WT inner retina, *p* < 0.001; rd10 outer retina *p* < 0.001; and rd10 inner retina *p* < 0.001).

**Figure 3:**
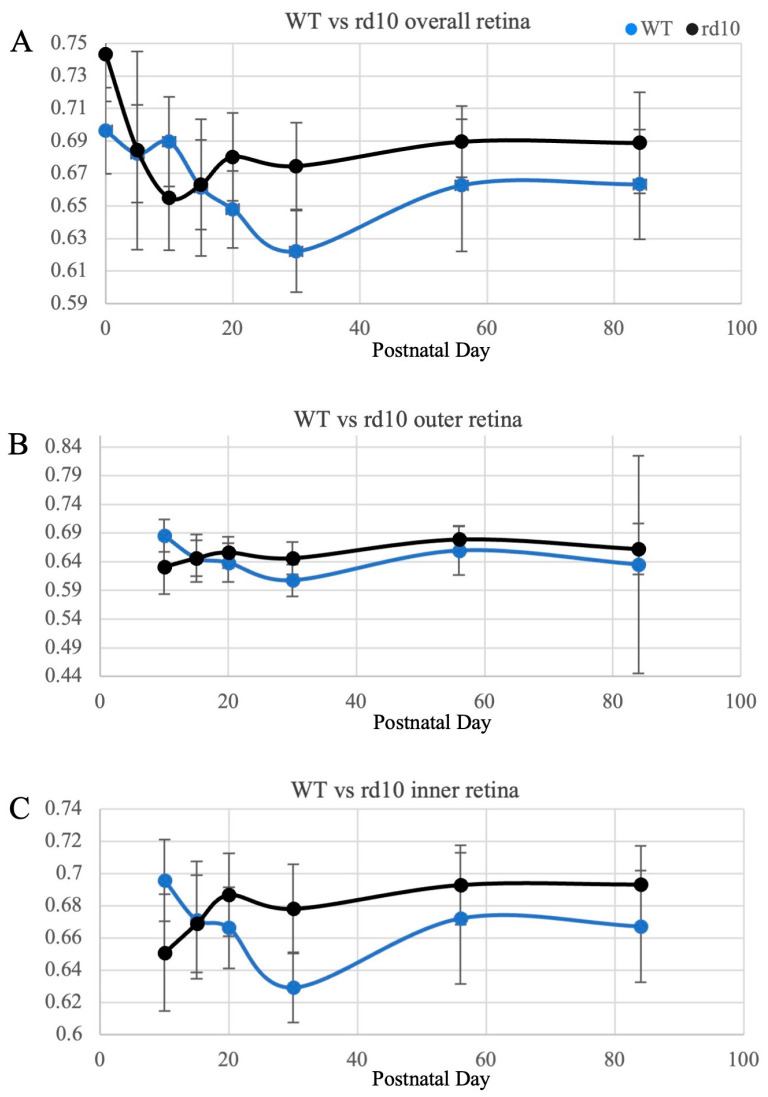
Percentage of bound NAD(P)H in the wild-type (WT) and rd10 mice retina over time. (**A**) Overall retina. (**B**) Outer retina. (**C**) Inner retina. Note that for the outer and inner retinas, the measurements started at P10. The rd10 retinas also showed higher percentages of bound NAD(P)H relative to their WT counterparts at almost all time points.

Overall, both WT and rd10 mice exhibited an initial decrease in OXPHOS, determined by the percentage of bound NAD(P)H, followed by a rise and gradual plateauing. In the rd10 mice, this trend was accelerated, with their percentages decreasing until P10, as opposed to P30 in WT, and then rising again till their percentages plateaued (Figure 3). In addition to analyzing the overall retina, we performed the analysis for the outer and inner retinas separately from P10 when the outer and inner retinas could be differentiated from each other (Figure 3). Both the outer and inner retinas follow the same trend as the overall retina in both the WT and rd10 mice. The WT and rd10 mice were overall significantly different from each other at each time point, with the rd10 mice showing noticeably increased OXPHOS relative to their WT counterparts at almost all times (Table 1).

**Figure 4:**
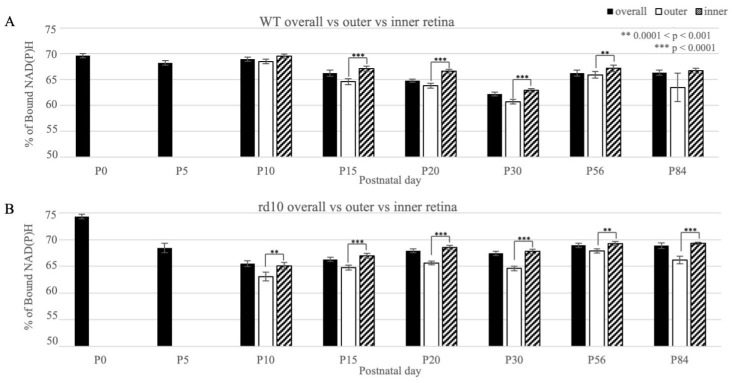
Percentage of bound NAD(P)H in the wild-type (WT) and rd10 mice retinas, comparing the overall, outer, and inner retina measurements. Measurements for the outer and inner retinas started at P10. (**A**) WT mice. (**B**) rd10 mice. Statistically significant differences between the outer and inner retinas are indicated above the bars, with more asterisks (*) denoting higher levels of significance (** 0.0001 < *p* < 0.001;*** *p* < 0.0001). There were higher percentages of bound NAD(P)H in the inner retina compared to the outer retina, a finding that was indicative of more OXPHOS and was mostly statistically significant across the timepoints in both strains.

Within discrete timepoints in both strains, there was also a significant difference in OXPHOS rates between the retinal layers, especially between the outer and inner retinas (Figure 4, Supplementary Table S1). Generally, there was greater OXPHOS in the inner retina relative to the outer retina, a finding that was statistically significant in the rd10 strain in every timepoint. In the WT mice, the same was true except at the P10 and P84 timepoints. Additional comparisons were made between the posterior, far-peripheral, and mid-peripheral zones, though generally, there was no significant difference found across these zones in either the overall, outer, or inner retina in either strain (Supplementary Figures S1 and S2 and Supplementary Tables S2 and S3).

## 4. Discussion

In the rd10 mice, retinal degeneration becomes histologically discernable around P16–18. Here, we demonstrate that there were significant differences in retinal metabolism between the WT and rd10 mice starting at birth. We used the percentage of bound NAD(P)H as a measurement of the metabolic state preserved upon fixation [14], with bound NAD(P)H indicating OXPHOS, and with the understanding that any relative changes between the bound and free states were being driven by the exchanging of electrons during OXPHOS and glycolysis. We showed that OXPHOS initially trended downwards in both the WT and rd10 mice. In the WT mice, the trough was at P30, and in the rd10 mice, the trough was reached sooner at P10. For both strains, bound NAD(P)H percentages rose and eventually plateaued, showing that over time, more OXPHOS was used compared to glycolysis. As consistent with Kooragayala et al.’s findings in P30 rd1 and rd10 mice, OXPHOS (and by proxy respiration) was shown to be elevated in the setting of significant retinal degeneration in almost all time points compared to the WT mice [24].

In prior studies, young retinas were observed to use glycolysis for biosynthesis rather than OXPHOS during proliferation, which is also seen in both normal and cancerous cells [25,26,27,28]. Aerobic glycolysis, although less energy efficient than OXPHOS, may provide the necessary building blocks for retinal growth. In proliferating cells, there are growth factor signals to promote early glycolytic pathway utilization. During these early glycolytic steps, growth factors inhibit later steps that would otherwise lead to cellular respiration and OXPHOS and instead force the glycolytic intermediates towards biosynthetic pathways. Through these pathways, macromolecules necessary for cellular proliferation are created, leading to downstream growth and development. As the retina differentiates and development continues, more oxygen is consumed, and there is a resultant shift towards OXPHOS over time. It is likely that such a phenomenon is reflected in our data as well, with the WT and rd10 mice retinas showing initially decreasing OXPHOS from P0 to P30 and from P0 to P10, respectively (likely in direct exchange for glycolysis), following which OXPHOS later increases and then plateaus.

In our data, WT and rd10 retinas were confirmed to remain structurally the same throughout the first 15 days of development [3,4,29]. As per Samardzija et al., and as confirmed in our results, after P15, WT photoreceptor outer segments continue growing till P28, following which their size stays relatively stable [4]. In our rd10 mice and those in the literature, after P15, photoreceptor outer segments visibly degenerated and almost completely disappeared by P28.

Based on the mechanisms previously discussed and the established structural–developmental timelines of the WT and rd10 mice, we speculate that in WT retinas, increased glycolysis is used to develop the photoreceptors until the outer segments have grown to their full size, roughly around P30. With fewer biosynthetic demands and more oxygen demand from the developed photoreceptors, there is increased OXPHOS, which eventually reaches a steady rate throughout adulthood. In rd10 mice, glycolysis is utilized more until around P15, where there are likely underlying signals to decrease biosynthesis or the signals for degeneration are stronger than those to proliferate. Without the need for continued proliferation, there is less need for glycolysis, and so, to provide the retina with energy the most efficiently, the eye turns to rely more on OXPHOS.

In terms of comparing the retinal layers (outer versus inner), we noted freer NAD(P)H, or more glycolysis, in the outer retina relative to the inner retina. Our findings were consistent with our previous study and Browne and colleagues’ work in human organoids, which showed more glycolysis in the outer layers and more OXPHOS in the inner layers of the organoids [10,14]. We also compared the posterior, mid-peripheral, and peripheral zones. Though there were some significant differences between these zones at certain time points, the zones did not show any significant patterns as a whole.

The limitations of our study included imaging fixed tissues, for which a previous study showed increased fluorescence lifetimes in certain fixatives [30]. However, in that study, the fixative time was not mentioned. Moreover, we used the same tissue preparation procedure for both the WT and rd10 mice.

In summary, our study compared the metabolic states and structures of WT and rd10 retinas throughout their first three months of life. We found that there was a trend towards decreased OXPHOS and increased glycolysis in the first few weeks of life in both the WT and rd10 mice, which gradually reversed and plateaued. In addition, we confirmed that the inner retina showed significantly more OXPHOS than the outer retina and that more centralized zones did not differ significantly in terms of metabolism versus more peripheral zones. In conclusion, these early developmental differences between the WT and rd10 mouse retinas may provide deeper insights into the cellular underpinnings and dysfunction of inherited retinal dystrophies and may facilitate future treatment development.

## Figures and Tables

**Table 1:** Average bound NAD(P)H percentages of the wild-type (WT) and rd10 mice for overall, outer, and inner retinas over time. Measurements of the outer and inner retinas started at P10. To account for multiple comparisons, we applied a Bonferroni correction by dividing the desired alpha level of 0.05 by the number of comparisons being made. All timepoints showed significant differences between the WT and rd10 mice, except for P5 and P15.

Retinal Region	Postnatal Day	WT	rd10	*p*-Value
Overall retina	0	69.6	74.3	<0.0001
	5	68.2	68.4	0.8417
	10	69.0	65.5	0.0001
	15	66.1	66.3	0.6294
	20	64.8	68.0	<0.0001
	30	62.2	67.5	<0.0001
	56	66.3	69.0	0.0002
	84	66.3	68.9	<0.0001
Outer retina	10	68.5	63.1	<0.0001
	15	64.6	64.7	0.5458
	20	63.8	65.6	0.0032
	30	60.7	64.6	0.0006
	56	65.9	67.9	0.0214
	84	63.5	66.2	0.3601
Inner retina	10	69.6	65.1	<0.0001
	15	67.1	67.0	0.9240
	20	66.6	68.6	0.0002
	30	62.9	67.8	<0.0001
	56	67.2	69.3	0.0061
	84	66.7	69.3	<0.0001

## Data Availability

The data presented in this study are available in the Supplementary Materials and within the article.

## Supplementary Materials

The following supporting information can be downloaded at: https://www.mdpi.com/article/10.3390/cimb46010039/s1, Figure S1: Percentage of bound NAD(P)H in wild-type (WT) mice, comparing the posterior, mid-peripheral, and far-peripheral zones. (A) Retinal zone comparison of the overall retina. (B) Retinal zone comparison of the outer retina. (C) Retinal zone comparison of the inner retina. Measurements for the latter two layers started at P10. Statistically significant differences between retinal zones are indicated with asterisks above the bars (*). Generally, there was no significant difference between the retinal zones of WT mice; Figure S2: Percentage of bound NAD(P)H in rd10 mice, comparing the posterior, mid-peripheral, and far-peripheral zones. (A) Retinal zone comparison of the overall retina. (B) Retinal zone comparison of the outer retina. (C) Retinal zone comparison of the inner retina. Measurements for the latter two layers started at P10. Statistically significant differences between retinal zones are indicated with asterisks above the bars on the left (*). Generally, there was no significant difference between the retinal zones of rd10 mice; Table S1: Average bound NAD(P)H percentages of wild-type (WT) and rd10 retinas for each timepoint, comparing the overall (*n* = 6–9), outer (*n* = 6–9), and inner (*n* = 6–9) retinas with *p*-values between the outer and inner retinas. The number of eyes that were analyzed is also listed below; Table S2: Average bound NAD(P)H percentages of wild-type (WT) retinas, comparing across timepoints the posterior (*n* = 6–9), mid-peripheral (*n* = 6–9), and far-peripheral (*n* = 6–9) zones; Table S3: Average bound NAD(P)H percentages of rd10 retinas, comparing across timepoints the posterior (*n* = 6–9), mid-peripheral (*n* = 6–9), and far-peripheral (*n* = 6–9) zones.
