# Supplementary material for: Comparison of Retinal Metabolic Activity and Structural Development between rd10 Mice and Normal Mice Using Multiphoton Fluorescence Lifetime Imaging Microscopy
Source: Curr Issues Mol Biol. 2024 Jan 6;46(1):612–20. doi: 10.3390/cimb46010039 (PMC10813981; doi:10.3390/cimb46010039)
Supplement: Supplementary file 1 [file cimb-46-00039-s001.zip › cimb-2794154-supplementary.pdf]

Supplementary Materials

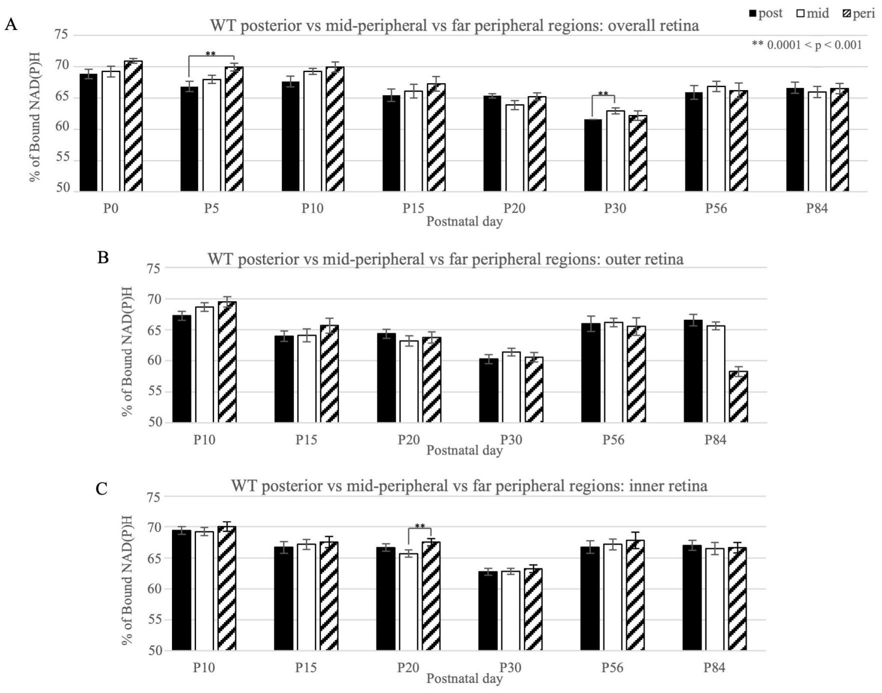

Figure S1. Percentage of bound NAD(P)H in wild-type (WT) mice, comparing the posterior, mid-peripheral, and far peripheral zones. (**A**) Retinal zone comparison for overall retina. (**B**) Retinal zone comparison for outer retina. (**C**) Retinal zone comparison for inner retina. Measurements for the latter two layers started at P10. Statistically significant differences between retinal zones are indicated with asterisks above the bars (*). Generally, there was no significant difference between retinal zones in WT.

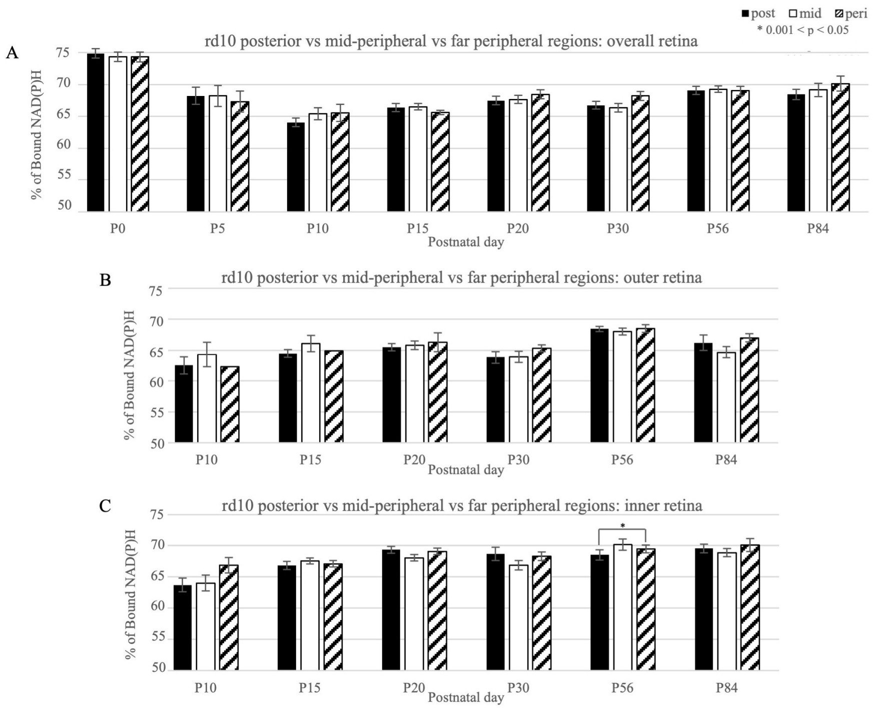

Figure S2. Percentage of bound NAD(P)H in rd10 mice, comparing the posterior, mid-peripheral, and far peripheral zones. (**A**) Retinal zone comparison for the overall retina. (**B**) Retinal zone comparison for the outer retina. (**C**) Retinal zone comparison for the inner retina. Measurements for the latter two layers started at P10. Statistically significant differences between retinal zones are indicated with asterisks above the bars on the left (*). Generally, there was no significant difference between retinal zones in rd10.

Table S1. Average bound NAD(P)H percentages of wild-type (WT) and rd10 retinas for each timepoint, comparing the overall (n = 6–9), outer (n = 6–9), and inner (n = 6–9) retina with p-values between outer and inner retina. Number of eyes analyzed is also listed below.

Strain	Postnatal Day	Overall retina	Outer retina	Inner retina	*p*-value
Wild-type	0	69.6			
	5	68.2			
	10	69.0	68.5	69.6	0.0586
	15	66.3	64.6	67.1	<0.0001
	20	64.8	63.8	66.6	<0.0001
	30	62.2	60.7	62.9	<0.0001
	56	66.3	65.9	67.2	0.00132
	84	66.3	63.9	66.7	0.245
rd10	0	74.3			
	5	68.4			
	10	65.5	63.1	65.1	0.00118
	15	66.3	64.7	67.0	<0.0001
	20	67.9	65.6	68.6	<0.0001
	30	67.5	64.6	67.8	<0.0001
	56	69.0	67.9	69.3	0.00370
	84	68.9	66.2	69.3	<0.0001

Table S2. Average bound NAD(P)H percentages of wild-type (WT) retinas, comparing across timepoints the posterior (n = 6–9), mid-peripheral (n = 6–9), and far peripheral (n = 6–9) zones.

Retinal Region	Postnatal Day	Posterior	Mid-peripheral	Far peripheral
Overall Retina	0	68.8	69.2	70.9
	5	66.8	68.0	69.9
	10	67.6	69.2	69.9
	15	65.5	66.1	67.2
	20	65.3	63.9	65.2
	30	61.6	62.9	62.2
	56	65.9	66.8	66.1
	84	66.6	65.9	66.5
Outer Retina	10	67.3	68.7	69.5
	15	64.0	64.1	65.7
	20	64.4	63.2	63.8
	30	60.2	61.4	60.6
	56	66.0	66.2	65.5
	84	66.6	65.6	58.3
Inner Retina	10	69.4	69.2	70.1
	15	66.7	67.2	67.6
	20	66.6	65.7	67.5
	30	62.7	62.8	63.2
	56	66.7	67.2	67.8
	84	67.0	66.5	66.6

Table S3. Average bound NAD(P)H percentages of rd10 retinas, comparing across timepoints the posterior (n = 6–9), mid-peripheral (n = 6–9), and far peripheral (n = 6–9) zones.

Retinal Region	Postnatal Day	Posterior	Mid-peripheral	Far peripheral
Overall Retina	0	74.9	74.3	74.3
	5	68.2	68.2	67.3
	10	64.1	65.4	65.6
	15	66.4	66.5	65.6
	20	67.5	67.7	68.5
	30	66.8	66.4	68.2
	56	69.1	69.3	69.0
	84	68.5	69.2	70.2
Outer Retina	10	62.5	64.3	62.3
	15	64.5	66.1	64.9
	20	65.5	65.8	66.3
	30	63.8	63.9	65.3
	56	68.4	68.0	68.5
	84	66.2	64.7	67.0
Inner Retina	10	63.7	64.0	66.9
	15	66.8	67.5	67.1
	20	69.3	68.0	69.0
	30	68.6	66.8	68.3
	56	68.5	70.1	69.4
	84	69.5	68.9	70.1
